# Visual Reliance in Severe Hearing Loss: Visual Evoked Potentials (VEPs) Study

**DOI:** 10.3390/audiolres15010003

**Published:** 2025-01-13

**Authors:** Takwa Gabr, Ahmed Hashem, Sherihan Rezk Ahmed, Mohamed G. Zeinhom

**Affiliations:** 1 Audiovestibular Medicine Unit, ORL Departement, Faculty of Medicine, Kafrelsheikh University, Kafr Elsheikh 33516, Egypt; 2 Ophthalmology Department, Kafrelsheikh University Hospitals, Kafr Elsheikh 33516, Egypt; ahmed_hashiem@med.kfs.edu.eg; 3 Neurology Department, Kafrelsheikh University Hospitals, Kafr Elsheikh 33516, Egypt; sherihanrezk2016@gmail.com (S.R.A.); mohamed_gomaa@med.kfs.edu.eg (M.G.Z.)

**Keywords:** severe hearing loss, cochlear implants, visual evoked potentials, visual cues

## Abstract

Peripheral hearing loss is associated with the cross-modal re-organization of the auditory cortex, which can occur in both pre- and post-lingual deaf cases. Background/Objectives: Whether to rely on the visual cues in cases with severe hearing loss with adequate amplification is a matter of debate. So, this study aims to study visual evoked potentials (VEPs) in children with severe or profound HL, whether fitted with HAs or CIs. Methods: This study included three groups of children matched in age and gender: normal hearing, children with hearing thresholds >70 dBHL and fitted with power HAs, and children fitted with CIs. All cases were subjected to pure tone audiometry (aided and unaided), speech discrimination scores, ophthalmic examinations, and visual evoked potentials (VEPs). Results: SD% scores significantly improved with the use of VCs in both CI and HL groups, and a significantly higher P100 amplitude of VEPs in both CI and HL groups (more in children fitted with CIs). Conclusions: Cross-modal reorganization in severe degrees of HL is of great benefit whether they are fitted with HAs or CIs.

## 1. Introduction

Orientation with the surrounding environment depends on the multisensory inputs and the coherent ability of our cognitive system to efficiently use and integrate such variable sensory inputs [1]. Hearing is an essential component of human beings’ learning of language and speech, the development of cognitive skills, and the provision of a good estimation of non-visible stimuli [2,3]. Hearing impairment is invisible, and many patients suffer in silence. The situation is more drastic in infants and young children since it hinders proper language acquisition with poor personal communication skills, in addition to social exclusion, loneliness, and dissatisfaction [4,5]. The lack of auditory input has a central plastic effect where the auditory cortex is reorganized and becomes stimulated by other modalities like vision and sensorimotor stimuli. In terms of visual stimulation of the auditory areas, many authors described this neuro-physiological phenomenon as “visual cross-modal reorganization”, where the visual cortex will utilize the auditory cortical regions for visual processing [6]. This cross-modal reorganization occurs in pre- and post-lingual deaf cases and is also noticed after using cochlear implants (CIs).

The early use of devices like hearing aids (HAs) or cochlear implants (CIs) is advantageous in the enhancement of hearing abilities [7]. However, there is still a problem in different aspects of speech processing. In other words, they may have slow processing of spoken language, which in turn has negative consequences for children’s academic achievement and psychosocial well-being. Those children have problems following conversations and miss essential parts of the spoken messages with much psychological stress due to their increased effort to comprehend different speech sounds. This will be associated with the depletion of cognitive resources with subsequent harmful effects on memory, attention, and learning [8,9].

One of the compensatory mechanisms used to compensate for the reduction in the processing speed is using visual cues (VCs). These cues include the observation of speakers’ facial movements and the head and eyebrow movements, where all these movements provide non-auditory cues to identify phonemes and prosodic structures [10,11]. They significantly affect the accuracy and speed of speech perception in various circumstances in both normal hearing or hearing-impaired adults and children in quiet, noisy listening conditions or degraded speech signals [12]. Similar improvement was also found in children using HAs or CIs which show benefits for accuracy when listening both in noise and in quiet [13]. The onset of hearing loss and its severity are crucial in determining the benefit of visual cues, whereas cases with early onset of HL and a more severe degree of HL are more likely to benefit from VCs [14].

Functional magnetic resonance imaging (fMRI) studies revealed the activation of the primary auditory areas of early deafened subjects in response to auditory stimulation. This suggested that early HL can induce visual stimuli processing within the auditory cortex, where the visual modality compensates for missed auditory inputs in degraded speech signals [15]. In previous work, Gabr et al. [16] reported a stronger response to VEPs in children fitted with CIs than in normal hearing children. This study assumes that VCs are stronger in pre-lingual deafened children with severe degrees of HL than in children with normal hearing (NH) or those fitted with CIs. We also believe that HL can initiate cortical organization in both the visual and auditory cortices. Studying VEPs in pre-lingual deafened children with severe degrees of HL did not receive much attention, and we assume it could be better than other children (NH or those with CIs) due to their greater dependence on the VC, which will be highlighted in this study.

## 2. Aim of the Work

This work is designed to study visual evoked potentials (VEPs) in children with severe or profound degrees of HL compared to normal hearing children and those fitted with CIs. It also aims to study any possible relationship between speech perception and the use of vision as an additional sensory input during auditory stimulation

## 3. Materials and Methods

Sixty-eight children were recruited for this work, matched in age and gender. They were divided into three groups: 22 normal hearing children (NH), 26 children fitted with CIs (CIs), in addition to 20 children with hearing thresholds >70 dBHL (HL), and their ages ranged between 5 and 16 years.

The CI group had pre-lingual onset of hearing loss (before the age of 2 years) and they were appropriately fitted with HAs according to their hearing thresholds in addition to a proper rehabilitation program for at least 6 ms. They were referred for CI due to the limited progress in the rehabilitation once they had fulfilled the other National Health Insurance CI Program selection criteria. As for the HL group, they also had a pre-lingual onset of HL, which was of a severe degree. They were also fitted with HAs according to their hearing thresholds and involved in rehabilitation sessions. However, their families refused to go through a CI program due to an improper experience with other siblings or refusal of the concept.

All children participating in this study were selected from the Audiovestibular Unit of the Otolaryngology Department at Kafrelsheikh University Hospitals. The examiners clarified the test procedure to the parents, and their consent was obtained afterward. This study adhered to the Code of Ethics established by the World Medical Association (2013) [17] and the Ethical Committee of Kafrelsheikh University with an approval number of KFSIRB200-274. The inclusion criteria of the study group included children aged between five and sixteen years who were appropriately and regularly fitted with HAs or CIs and had adequate maintenance of both devices. All children must have no visual problems.

Exclusion criteria included un-cooperative children such as those with mental retardation, behavioral or developmental disorders, irregular use or inadequate maintenance of HAs or CIs, improper rehabilitation therapy, or those with visual problems.

## 4. All Children Were Submitted to the Following

1. Thorough otological and audiological history.
2. Hearing evaluation using pure tone audiometry (along the frequency range of 250–8000 Hz for air conduction and 500–4000 Hz for bone conduction) and speech audiometry using speech materials specially designed for children. The technique of hearing evaluation was dependent on the child’s cooperation, either play audiometry or voluntary thresholds. Speech audiometry included speech reception threshold (SRT) and discrimination scores (SD%), conducted with and without VCs. Both PTA and speech audiometry were conducted using Interacoustics AD629 (Middelfart, Denmark).
3. Assessment of middle ear function through immittancemetry using Interacoustics AT235 (Middelfart, Denmark).
4. Check up on CIs and HAs for both HL and CI groups, followed by sound field examination using warble tones (250–4000 Hz) and speech materials. The Arabic version of 50-PB-Kg lists were used to assess the SD% at 40 dBSL (re-aided SRTs).
5. The ophthalmic examinations included evaluating the child’s medical and family history of ocular conditions. They also involved observing for external ocular abnormalities, examining the pupil and corneal light reflexes, assessing the range of ocular movements, visual acuity, and examining the anterior segment and posterior segment.
6. Visual evoked potentials (VEPs) were recorded using a reversing white and black checkerboard with a mean luminance of 70 cd/m^2^ and a contrast close to 100%. The stimuli were displayed on a monitor with a central red dot for fixation. The electrode montage was set at 4 cm above the inion for the active electrode, at the forehead (Fz) for the reference electrode, and at the lower forehead (Fpz) for the ground electrode. For more details, see Gabr et al. [16]. Children were instructed to fixate on the central red dot in the checkerboard. The VEP analysis revealed three peaks: N75, P100, and N145. To ensure reproducibility, three responses were recorded where both P100 latency and P100-N145 peak-to-peak amplitude were calculated in each run. Recording of VEPs was by using Nihon Kohden MEB-2300 Neuropack X1 (Tokyo, Japan) and the stimuli were displayed on a monitor Model CPD-3214 (Samsung, Beijing, China)

Statistical analysis was conducted using the IBM SPSS software package, version 20.0 (Armonk, NY, USA: IBM Corp.). Data analysis was conducted depending on their distribution, as determined by the Shapiro–Wilk test. Numerical data were described as median and interquartile range (IQR) while categorical data were reported using numbers and percentages. The Mann–Whitney U test was used to compare the abnormally distributed numerical data between two groups, while the Kruskal–Wallis test was used to compare the abnormally distributed numerical data of the three groups. Additionally, the Bonferroni post-hoc test was used for pair-wise comparisons. Correlating the categorical was performed using Pearson’s Chi-squares, and detecting the association between numerals was performed using the Spearman correlation. All the data were included in our study, and all statistical analyses were two-sided. Differences with a *p*-value of less than 0.05 were considered statistically significant.

## 5. Results

The NH (control) group consisted of 22 children (13 males and 9 females) with a mean age of 10.9 ± 3.4 years. The CI group consisted of 26 children (16 males and 10 females) with a mean age of 10.02 ± 3.7 years, and the HL group consisted of 20 children (12 males and 8 females) with a mean age of 11.5 ± 2.5 years. There was no significant difference between the three groups regarding age or gender (*p* > 0.05).

Hearing evaluation was conducted in the three groups. The NH group showed a bilateral normal peripheral along the whole frequency range for air conduction and bone conduction thresholds, normal middle ear function, and normal and consistent acoustic reflex thresholds with PTA. SRTs were consistent with PTA, and SD% were excellent in both ears (100 ± 0.00%).

**Figure 1:**
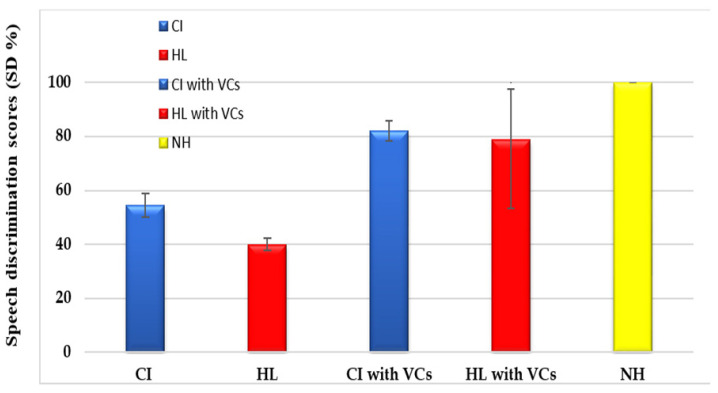
Comparison of the speech discrimination scores (SD%) with and without VCs in the cochlear implant (CI, 26 cases), hearing loss (HL, 20 cases), and normal hearing (NH, 22 cases) groups.

Regarding the CI group, the mean age for diagnosis of HL was 1.9 ± 0.9 years, where children were appropriately fitted with HAs in both ears (according to their hearing thresholds) for at least 6 ms and engaged in a properly designed rehabilitation program before being referred to the CI program. The mean age of CI surgery was 2.4 ± 0.3 years. Their aided audiological thresholds were satisfactory (≤30 dBHL) along the frequency range of 250–4000 Hz. The aided speech reception thresholds (SRTs) were within the normal range (27.307 ± 5.8 dB). The speech discrimination scores (SD%) were fair (54.46 ± 23.6%) and improved significantly with the use of VCs to 82.15 ± 3.8% (*p* < 0.0001) (Table 1, Figure 1).

Regarding the HL group, the mean age of diagnosing HL was 1.8 ± 0.6 years. As in the CI group, they were appropriately fitted with HAs (according to their hearing thresholds) in both ears and enrolled in properly designed rehabilitation programs. They were not referred to CI programs due to the family’s satisfaction with HA results or refusal to proceed with CI. Their mean PTA in both the right and left ears were 81.16 ± 11.64 dB and 81.96 ± 10.6 dB, respectively, with no significant difference. Their mean aided thresholds were 34 ± 10.54 dB and 37.2 ± 15.32 dB in both the right and left ears, respectively, with no significant difference. Speech audiometry showed no difference between the right and left ears regarding SRTs or SD% (with and without visual cues). In each ear, the use of VCs resulted in a significant improvement in the SD% either in unaided or aided conditions (Table 2; Figure 1).

Further, the comparison between the CI and HL groups showed no significant difference in SD% with VCs (82.15 ± 3.8% and 78.8 ± 25.4% respectively, *p* = 0.558).

**Figure 2:**
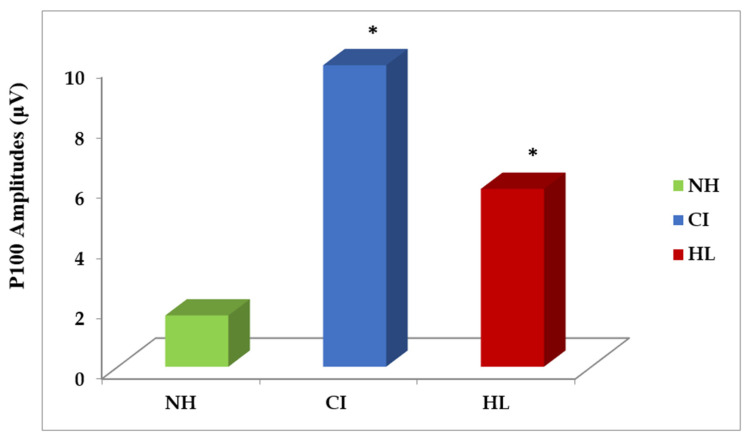
Amplitude of P100 in the normal hearing (NH, 22 cases), cochlear implant (CI, 26 cases), and hearing loss (HL, 20 cases) groups. * *p* is significant.

Ophthalmic examinations were normal in the three groups. The results of the VEPs revealed three peaks named N75, P100, and N145 according to their latencies. In each group, the comparison between the right and left eye recordings of VEPs revealed no significant differences. So, data from both sides were collected together for further analysis. The comparison of VEP latencies of N75, P100, and N145 between the three groups showed no significant difference. However, the amplitude of P100 showed a significant difference between the three groups. The post-hoc Bonferroni test revealed that both the CI and HL groups had a significantly higher P100 amplitude than the NH group. Both the CI and HL groups showed significantly higher amplitude in children fitted with CIs than in the HL group (Table 3 and Table 4; Figure 2).

Correlation was studied between SD% with the use of visual cues and the results of VEP recording. In the CI group, there was no significant correlation between latencies or amplitudes of VEPs and the SD% with VCs. However, the SD% with VCs in the HL group showed a significant positive correlation with P100 amplitude, meaning that as the SD% with VCs increases, the P100 amplitude increases (Table 5).

## 6. Discussion

With auditory deprivation, especially pre-lingual onset, the auditory cortex becomes vulnerable to recruitment by other sensory modalities (visual and somatosensory). This is called cross-modal reorganization of the auditory cortex, with enhancement in those functions that require both the visual and auditory functions, such as localization, movement, or change detection [18,19].

This study included three groups of children aged 5–16 with no significant difference in age or gender (*p* > 0.05). In the CI group, aided hearing and aided speech reception thresholds were satisfactory and within normal range. Regarding the speech discrimination scores, fair scores were obtained that significantly improved with VCs. Similarly, children in the HL group showed significantly improved performance with their HAs regarding their aided thresholds or aided speech audiometry results. They also showed significantly better speech reception thresholds and discrimination scores using VCs. These finding revealed the devastating effect of early onset HL on the development of auditory abilities in children due to limited auditory input. This effect occurs as a result of disruption in dendritic arborization and desynchronization of activity between cortical layers with subsequent plastic changes [20,21]. Additionally, the auditory cortex and the superior temporal gyrus showed a reduction in white matter in deaf individuals compared to those with normal hearing [22]. So, cortical areas are left unstimulated properly by sounds and become vulnerable to invasion by other sensory modalities like vision or somatosensory, known as cortical reorganization [23]. The improvement of speech discrimination scores with the use of VCs suggested that the dependence of cases with HL on the remaining senses facilitates their daily activities [24].

The loss of hearing also leads to changes in the attention process, with a redistribution of attentional resources at the central and peripheral levels [25], where the middle temporal (MT) and the middle superior temporal (MST) areas are well-recognized sites of adaptation following early onset HL. Both areas involved in visual motion processing showed increased activation following HL [26]. Moreover, animal studies showed a reorganization of the primary auditory cortex neurons to process visual information without auditory input [27,28]. This finding was supported by studying the blood-oxygen-level-dependent (BOLD) activities in the auditory cortex of deaf humans, which showed changes in response to visual motion [22].

Animal studies also provided evidence of visual reorganization in the posterior auditory field, which became involved in visual localization instead of being involved in auditory localization in normal hearing animals [29,30].

With the restoration of hearing via CIs or HAs, the auditory cortical regions regain some of their responsiveness to auditory stimuli; however, it might not be wholly reversed due to the establishment of cortical reorganization. This explains the need for early hearing restoration in cases with early onset HL and its close connection with better speech perception outcomes [7]. However, those children still have problems with different aspects of speech processing, such as a slow rate of language processing, causing poor academic performance and increased listening efforts [31,32]. Effortful processing of sounds results in the depletion of cognitive resources, with a drastic effect on memory and learning, leading to easy fatigability and stress [9].

One of the available solutions to overcome this problem is the provision of additional visual cues, such as observing the facial movements and expressions of the speakers. Visual cues have been proven to be beneficial in children with normal hearing in terms of detection of speech, proper perception of degraded speech signals, or in the presence of noise [33]. The positive effect of visual inputs also extends to children with HL, whether using HAs or CIs in quiet and noisy situations and emphasizes the need for VCs even if the hearing was restored with HAs or CIs [34,35]. Following HL, two types of cortical reorganization occurred in the visual and auditory cortices where the auditory cortex became responsive to visual stimulation, and the visual cortex became responsive to sounds. Auditory cortex reorganization has a detrimental effect on sound processing due to auditory deprivation, leading to poor speech perception.

On the other hand, visual cortex reorganization has a beneficial effect where their stimulation (by VCs) and by sounds contribute significantly to better speech perception [36,37], as reflected by the improvement of SD% with the use of VCs in both the HL and CI groups. Some studies reported (Ex. [30,38,39]) that cross-modal plasticity following HL might be associated with enhanced performance in the remaining modalities, where those individuals with early onset of HL showed better than normal performance in tasks of visual–spatial localization or visual motion detection. Studies in congenitally deaf cats (CDCs) show supranormal performance in their visual localization and visual motion detection abilities compared to normal hearing cats [30]. Interestingly, it was found that central regions responsible for such supranormal visual performance were the posterior auditory field and the dorsal auditory cortex [19].

The VEP is an evoked potential used to assess the functional integrity of the visual system. In this study, we assume that the use of visual cues in children with more severe degrees of HL might contribute to better VEPs than age-matched normal hearing children. Results of the VEPs revealed that both children fitted with CIs and those with HL had better VEP response in terms of higher amplitude, which was highly significant than the normal hearing group. This indicated the visual reliance of those children on their visual inputs during the processing of speech sounds. All children in the CI and HL groups had severe degrees of HL, where one group was fitted with unilateral CIs, and the other was fitted with HAs. Both groups had combined pathology as a sequence of HL including a lack of surviving hair cells, with the possibility of the presence of cochlear dead regions, poor temporal and spectral resolution, recruitment, and the loss of auditory filter sharpness [40,41,42].

Children in the HL group were fitted with power HAs with adequate detection of sounds, as shown in their good-aided responses. However, with this severe degree of HL, there is an inadequate auditory processing related to sound distortion at high output levels of power HAs, where a broader cochlear region is stimulated, resulting in a further reduction in the accuracy of speech decoding. Moreover, they have poor spectral resolution that facilitates noise passage through the broad auditory filters and subsequent speech masking. Another factor is the impaired temporal resolution, which is thought to be responsible for the improper encoding of the timing of auditory inputs as reflected in poor speech-processing abilities in those patients [40,43]. All these factors could contribute to the use of visual cues to compensate for the degraded acoustic inputs through HAs.

Regarding the children with CIs, they have similar preoperative hearing thresholds as the HL group. However, the parents decided to go through CI surgery to have better outcomes. So, children with CIs are supposed to have similar pathological sequences of severe HL. They also suffer the less optimal quality of sounds provided through their CI, where hearing is restored but in a completely different way through electrical hearing. Sound provided by a CI is known to be spectrally degraded, which could impact their perception of more complex forms of sound, such as speech prosody and music [44]. CIs are designed to imitate normal cochlea and transmit high-frequency sounds to the basal cochlear region and low-frequency sounds to the apical cochlear region. However, there is a physical mismatch between the processed frequency (transmitted via CI electrodes) and the actual place along the basilar membrane due to variations in cochlear size, length of the electrode array, proximity to nerve fibers, or the insertion depth leading to place pitch mismatch [45]. Additionally, the limited frequency range delivered through CI (≈200–8500 Hz), the possibility of current spread, and channel interaction will further degrade the CI sound quality and contribute to poor pitch perception [46,47]. Additionally, CI children lack access to fine structures such as the low-frequency voice pitch available to HA users, and are more susceptible to noise [48]. This might explain their need for additional cues, such as VCs, than children with HL fitted with HAs. VEP data in this work revealed a higher P100 amplitude in the CI group than those with severe to profound HL and fitted with power HAs, suggesting that children with CIs are more likely to use vision under the same testing conditions than children with HL. This highlighted the value of using VCs in CI children to achieve better outcomes and improved sound perception, especially in noise, depending on their better multisensory integration [49].

An additional factor that could contribute to more visual reliance in CI children is that they were unilaterally fitted with CIs as the contralateral ear did not receive any amplification due to the non-use of HAs in that ear after CI surgery due to poor sound quality. Thus, we could consider them as having “single-sided deafness (SSD)” [within normal hearing in one ear provided through CI and severe to profound HL in the unaided ear]. However, the situation is worse than those with SSD as they are unilaterally dependent on the CIs that deliver sounds with limited spectral and temporal resolution and significant distortion compared to the natural acoustic hearing through their devices, as mentioned before [50]. The visual reliance of CI cases is prone to adaptation development to the degraded auditory input after prolonged use of the CI [51].

Correlation was studied between SD% with VCs and the results of VEPs recording in both CI and HL groups. Only a significant positive correlation existed between SD% with VCs and P100 amplitude in the HL group. This means that, as the SD% with VCs increases, the P100 amplitude increases, which indicates that visual inputs are essential contributors to the successful speech discrimination process.

This study highlighted the importance of visual cues for cases with HL, whether they are fitted with HAs or CIs, especially in adverse listening conditions such as noisy environments [52]. VCs significantly contribute to and are integrated with acoustic information during the processing of speech [53], in addition to playing a significant role in developing different cognitive skills [54]. The use of VCs was found to activate the left superior temporal areas in a similar way as the auditory inputs [55]. Their use, especially in the educational setting of HL students, is recommended to access information and participate in the discussion and development of their skills [56,57] with gradual improvement of the cognitive and verbal skills [58], understanding of the new information, in addition to better interaction in discussion and retention of knowledge [59]. The efficient integration of both auditory and visual inputs is essential for efficiently exploring the surrounds [60].

However, using visual cues showed significant variability among different cases with HL due to several factors. *First*, the subjects’ central abilities where factors such as attention, the status of the working memory, and lip-reading skill contribute to greater audio–visual benefits [61]. *Second*, the characteristics of the HL, such as its early onset (pre-lingual), longer duration, and the greater severity of HL, are associated with better use of VCs [14]. *Third*, the duration and the proper use of hearing devices (HAs or CIs) have a positive effect [62]. The configuration of the hearing devices is essential, as children with unilateral HL are more likely to benefit from VCs than those with bilateral HL. This is because they have better access to the auditory inputs through their normal hearing ear, which allows for a more effective use of VCs and better processing speed that facilitates multimodal integration. *Fourth*, rehabilitation therapy that emphasizes the use of visual information usually contributes to better performance with the use of VCs [50]. *Lastly*, the task used to evaluate the efficacy of the VCs and its difficulty where tasks that used poorer SNR showed increased benefits from VCs. Additionally, tasks that required processing efforts, such as pupillometry, showed that children with poor phonological skills have difficulty in monitoring their phonemes with limited benefits from VCs [33].

The effect reorganization of the auditory system following HL is quite evident. It might involve different neural mechanisms: unmasking of silent inputs, preservation of transient connections, sprouting of axons, or a combination [63]. However, there is a lack of evidence to support these mechanisms [64,65], where only a small percentage of new connections to non-auditory areas were found [19]. CDC studies provided evidence for such findings where the small percentage of new connections cannot account for behavioral change, the supranormal detection of visual motion, and the enhanced visual localization abilities in those cats. Rather, they may represent an experiential modification of projections preserved in congenital deafness [66,67,68].

An alternate explanation of cross-modal plasticity was the reorganization of the auditory cortex at the functional level without actual plastic changes [65,69]. One of these functional organization sites is the brainstem and its nuclei (dorsal cochlear nucleus, the inferior colliculus and the trigeminal nucleus). The cochlear nucleus (CN) is the first place in the ascending auditory pathway and any functional changes within that nucleus (as a result of HL) will affect the whole auditory pathway, including the auditory cortex. For example, the trigeminal and cervical somatosensory regions will be represented in the deafened auditory cortex on both sides. So, the brainstem plays a vital role in cortical cross-modal reorganization (not plasticity) [70]. In deafness, eliminating the auditory inputs initiates a homeostatic plasticity that adapts the neural firing and induces synaptic changes that affect the balance between excitatory–inhibitory neurons to generate action potentials. Additionally, the sensitivity to the remaining sensory inputs will increase and cannot fully compensate for the absent auditory input. However, they have a role in activating the deprived auditory cortex [71,72]. With severe degrees of HL, cross-modal reorganization affects only the multimodal functions that the auditory system shares with other sensory systems and induces a behavioral advantage and functional connectivity enhanced by synaptic plasticity. When the input to the auditory cortex is appropriately restored via hearing aids and/or cochlear implants, excitability is dynamically downregulated, reversing the cross-modal changes to some extent. The somatosensory cross-modal effects can be reversed more completely than the visual one [73]. This finding is consistent with the continued reliance on visual inputs for distorted auditory signals through a hearing aid or cochlear implants.

Limitations of this study include the small sample size and not considering individual factors in the interpretation of the VEPs. Additionally, there is no recording of other AEPs to make a correlation between VEPs and central auditory processing.

## 7. Conclusions

This study highlighted the occurrence of cross-mod-l reorganization in cases with severe degrees of HL, which are not entirely reversed with HAs or CIs. These visual abilities are subjected to individual factors such as onset, degree, laterality of HL, and configuration of the assistive hearing devices (HAs or CIs). VEP is an effective evoked potential for assessing visual system contribution to sensory integration in cases with HL. VEP recording in CI cases revealed their great visual reliance on visual cues in their lives to compensate for the degraded acoustic signals delivered via the CI.

## Figures and Tables

**Table 1:** Results of aided pure tone thresholds and speech audiometry in the CI group.

Aided PTA In CI Group (*n* = 26)							
250 Hz	500 Hz	1000 Hz	2000 Hz	4000 Hz	SRT	SD%	SD% with VC
26.15 ± 5.2	26.25 ± 6	25.58 ± 5.3	24.42 ± 5.3	25.5 ± 6.9	27.3 ± 5.8	54.46 ± 4.5	82.15 ± 3.8
						T = 4.38 *p* < 0.0001	

**Table 2:** Results of pure tone audiometry along the frequency range of 250–8000 Hz and speech audiometry in the HL group (N = 20).

	PTA in HL Group (N = 20)							
	250 Hz	500 Hz	1000 Hz	2000 Hz	4000 Hz	8000 Hz	PT Averages	Average Aided Thresholds
Right	64.25 ± 11.68	68.5 ± 9.6	79.25 ± 7.6	89.25 ± 12.6	89.5 ± 18.3	96.25 ± 12	81.16 ± 11.64	34 ± 10.54
Left	67.25 ± 13	69.75 ± 8.9	80.25 ± 8.6	87.5 ± 14.4	91.25 ± 20.4	95.8 ± 18.5	81.96 ± 10.6	37.2 ± 15.32
	t = −0.7677 *p* = 0.447	t = −0.427 *p* = 0.671	t = −0.389 *p* = 0.699	t = 0.409 *p* = 0.684	t = 0.285 *p* = 0.776	t = 0.091 *p* = 0.93	t = −0.227 *p* = 0.82	t = −0.796 *p* = 0.44
	**Speech Audiometry**							
	**SRTs**		**Unaided SD%**				**Aided SD%**	
	**Unaided**	**Aided**	**Without VCs**		**With VCs**		**Without VCs**	**With VCs**
	80 ± 15.08	34.25 ± 10.2	37 ± 24.9		63 ± 21.42		40 ± 2.25	78.8 ± 25.4
	Z = 11.24 *p* < 0.001		Z = −3.54 *p* < 0.001				Z = −4.75 *p* < 0.001	

PTA: pure tone audiometry; PT: pure tone; SRTs: speech reception thresholds; SD%: speech discrimination scores; VCs: visual cues; N: number of cases.

**Table 3:** The comparison of latencies and amplitude of different VFP components between the three groups.

VEPs		NH (N = 22)	CI (N = 26)	HL (N = 20)	Test Statistic	*p*-Value
Latency	N75	76.5 (72.6–88.7)	75 (71.9–78.9)	83.2 (72–103.5)	1.200	0.55
	P100	118.5 (116.7–119.9)	114 (110.9–121.2)	129 (113.9–157.3)	3.994	0.14
	N145	166.8 (160.7–170.4)	167.7 (154.5–177.3)	197.3 (146.4–238.8)	4.934	0.09
Amplitude	P100	1.7 (1–2.7)	10 (7.8–12.4)	5.9 (3.2–6.9)	37.445	<0.001 **

Median (IQR: interquartile range); HL: hearing loss group; CI: cochlear implant group; N: number of cases; ** *p* is significant.

**Table 4:** Post-hoc Bonferroni comparison between P100 amplitude of VEPs in the three groups.

Group	Test Statistic	*p*-Value
Normal-HL	11.780	0.001 **
Normal-CI	40.615	<0.001 **
HL-CI	11.896	0.001 **

** *p* is significant.

**Table 5:** Spearman correlation between SD% with VCs and VEPs in CI and HL groups.

CI group (N = 26)		r	*p*-Value
Correlation Between VEPs Components and SD% with VCs			
Latency	N75	0.098	0.634
	P100	0.1666	0.416
	N145	0.198	0.331
Amplitude	P100	0.018	0.98
**HL Group (N = 20)**		**r**	***p*-value**
**Correlation between VEPs components and SD% with VCs**			
Latency	N75	0.237	0.207
	P100	0.279	0.136
	N145	0.077	0.685
Amplitude	P100	0.565	0.001 **

** *p* is significant.

## Data Availability

Data is available upon request.
